# Responses of phenology, yield attributes, and yield of wheat varieties under different sowing times in Indo-Gangetic Plains

**DOI:** 10.3389/fpls.2023.1224334

**Published:** 2023-07-21

**Authors:** Abdus Sattar, Gangadhar Nanda, Gulab Singh, Ratnesh Kumar Jha, Santanu Kumar Bal

**Affiliations:** ^1^ Centre for Advance Studies on Climate Change, Dr. Rajendra Prasad Central Agricultural University, Pusa, India; ^2^ Department of Agronomy, Dr. Rajendra Prasad Central Agricultural University, Pusa, India; ^3^ AICRP on Agrometeorology, ICAR-Central Research Institute for Dryland Agriculture, Hyderabad, India

**Keywords:** wheat, phenology, yield, sowing window, heat stress, simulation, CERES-wheat

## Abstract

A field experiment with wheat was conducted at Pusa (25.98°N, 85.67°E, 52 m amsl), Bihar (middle Gangetic plains of India), to assess the responses of phenology, yield attributes, and yield to growing season temperature and heat stress. For this purpose, wheat was planted on five dates (viz., 15 November, 25 November, 5 December, 15 December, and 25 December) for three consecutive years (viz., 2014–2015, 2015–2016, and 2016–2017) with three prominent cultivars of the region (viz., RAU-3711, HD-2824, and HD-2733). Five dates of sowing represent different wheat-growing micro-environments as imposed by varying sowing dates encompassing the entire sowing window. The study observed the significant effect of sowing dates on phenophase duration. In general, with progress in the date of sowing, tiller initiation was delayed, while the reverse trend was observed for later growth phases. Sowing environments significantly influenced the number of effective tillers m−2. Average numbers of effective tillers (ET) m−2 for the wheat sown during 15–25 November were almost 11.6% higher than those of the 25 December sown crop. Grain filling duration (GFD) showed a declining trend with the advancement of sowing dates due to increased thermal load on the crop during the reproductive period. 15 November planted crop exhibited the highest GFD (47 days), which was shortened significantly beyond 25 November, signifying agrometeorological non-suitability of wheat sowing beyond this window. Wheat sown on 25 November recorded the highest grain yield (3.21 Mg ha−1), 48.61% higher than the 25 December sown crop due to the congenial thermal regime. In this context, we have identified optimal and sub-optimal conditions to escape heat stress for higher wheat productivity. Moreover, the sum of deviation of temperature from optimum thresholds, computed for sensitive growth phases (50% flowering to physiological maturity), helped us to identify heat stress and its impact on wheat. Genotype-by-environment (GGE) biplot analysis revealed that RAU-3711 was found to be the most stable cultivar. A decrease in the yield of wheat by 4.9% to 12.0%, sown during November, and 33.8% to 42.4%, sown during December, is predicted in 2050-51 and 2080-81, respectively, under RCP 4.5.

## 1 Introduction

Wheat is the most important staple food crop in the world. It also plays a vital role in food security for millions of people in India and occupies approximately 30.6 million hectares area with a total production of 98.5 million tonnes (FAO, 2017) contributing approximately 43% to the country’s granary (Bapuji Rao et al., 2015). India is the second largest producer of wheat after China with approximately 12% share in global wheat production. In recent years, rising temperature due to climate change has been a cause of concern for sustainable wheat production in India, more specifically over Indo-Gangetic Plains stretching a vast area from Punjab in the West and Bihar in the East. The concern is real regarding the negative effect of warming on the phenology and yield of crops (Xiao et al., 2012; Wang et al., 2013). Under such a situation, several adaptation measures, viz., wheat sowing by zero tillage, growing new heat-resistant cultivars, and changing planting schedules, are being taken up by policymakers and farmers. Wheat production in Indo-Gangetic Plains is vulnerable to short-term temperature extremes (Lobell et al., 2012). Prevalence of dry westerly wind and sudden rise of temperature during the fag end of the growing season tends to shorten the grain filling period (Garg et al., 2013; Sandhu et al., 2016; Vashisth et al., 2020) and negatively impact grain setting of wheat (Sattar and Srivastava, 2021). It significantly affects growth and photosynthetic efficiency (Wang et al., 2011) and consequently reduces biomass and productivity (Farooq et al., 2011). Grain number, which is determined from 30 days before flowering until shortly after flowering, and grain size, dependent on grain filling (Lobell et al., 2012), crop duration, and crop biomass, together tend to decide the final yield of the crop.

Phenology is an integral part of crop weather models, which is used to specify the appropriate time and rate of specific phasic development processes (Singh et al., 2001). In this context, studying crop phenology vis-à-vis thermal regime in the field and the integrated effect of weather on yield assumes significance to bring in proper resilience against the adverse impact of high temperature on crop growth. Varying dates of sowing expose the crop in a year to different temperatures during its growing period, which helps properly understand the response of phenology to ambient temperature (Vijaya et al., 2015). Xiao et al. (2013) showed that changes in the phenological phases of winter wheat are strongly related to temperature trends. Given the potential impacts of global warming on yield, the study of phenology assumes great importance due to its impacts on productivity and farming practices (Xiao et al., 2012; Wang et al., 2013). Temperature plays a great role in modifying the enzymatic functions of plants and causes a change in phenology, which is directly related to yield (Zhu et al., 2018).

Temperature-based agrometeorological indices, viz., growing degree day (GDD), helio-thermal unit (HTU), and photo-thermal units (PTU), have a direct relationship with the growth and yield of crops. Accordingly, these indices along with thermal efficiencies are important parts of understanding the responses of phenology and yield to growing season temperature. Heat stress manifested by the occurrence of significantly higher than normal temperatures for 15–25 days during the reproductive period of wheat in the *rabi* (winter) season of 2021–2022 caused a significant reduction of grain yield in India (Bal et al., 2022). Against the backdrop of the problem of terminal heat stress and variable wheat yield, we hypothesized that by optimizing sowing dates, wheat can be grown under a congenial thermal regime, thus offsetting the negative impacts.

Since temperature significantly influences wheat yield, it would be prudent to simulate future yield under a projected climatic scenario. Future wheat yield can be simulated by different crop simulation models. The CERES-Wheat model of Hoogenboom et al. (2019) is the most widely used crop growth model, and it is an effective tool to quantify the effects of cultivar, climate, soil, and management on wheat growth across the globe. It can be effectively used to simulate yield in the projected climate (Shen et al., 2022). Keeping in mind the above facts, an attempt has been made in this article to evaluate the responses of phenology and yield of wheat to growing season temperature and heat stress. A systematic study on this aspect for a region like the middle Gangetic Plains of eastern India appeared to be meager. In this context, the objectives of the investigation were set i) to quantify the impact of varying thermal regimes induced by varying sowing environments on crop phenology and yield ii) to optimize the exact sowing environment of wheat based on the response of phenology and yield to growing season thermal regime.

## 2 Materials and methods

### 2.1 Study location

The field experiment was carried out at the University Farm of Dr. Rajendra Prasad Central Agricultural University, Pusa (25.98°N, 85.67°E, 52 m), Bihar, located in the middle Gangetic plains of India.

### 2.2 Climate and soil

The region experiences a sub-humid subtropical monsoon climate. It has four major seasons, viz., summer (March–May), monsoon (June–September), post-monsoon (October–November), and winter (December–February). The average annual rainfall of the area is approximately 1,230 mm. Approximately 85% of rainfall occurs during the *kharif* (monsoon) season. May is the warmest summer month of the year with a daily maximum temperature of 37°C–41°C, while the coldest winter month is January with a daily minimum temperature of 5°C–8°C. December, January, and February are the main winter months in the region. Temperature decreases significantly from November, which becomes lowest in January. Increasing temperature from March onward heralds the commencement of the summer season. Locally, the period from 15 October to 15 March is called *rabi* season, in which important irrigated crops such as wheat, maize, potato, mustard, cauliflower, cabbage, and chickpea are grown.

The soil of the experimental field has sandy loam soil, which is the dominant soil textural class of the region. The physicochemical properties of the experimental soil are given in **Table 1**.

**Table 1:** Physio-chemical properties of soils of the experimental field.

S. no.	Specifics/parameters	Initial value
1.	Sand (%)	55.94
2.	Silt (%)	31.85
3.	Clay (%)	12.20
4.	Textural class	Sandy loam
5.	Electrical conductivity (dS m^−1^)	2.94
6.	pH	8.28
7.	Organic carbon (%)	0.47
8.	Available N (kg ha^−1^)	241.0
9.	Available P (kg ha^−1^)	17.18
10.	Available K(kg ha^−1^)	160.0
11.	Available Zn (kg ha^−1^)	1.33

### 2.3 Methodology

The experiment was conducted over three wheat growing seasons, viz., *rabi* (winter) seasons of 2014–2015, 2015–2016, and 2016–2017. The crop was planted on five dates every year (viz., 15 November (D1), 25 November (D2), 5 December (D3), 15 December (D4), and 25 December (D5)) with three prominent cultivars (viz., RAU-3711, HD-2824, and HD-2733) of the region in factorial randomized block design with three replications. The sowing was staggered to impose heat stress on the crop at critical growth phases. Recommended package of practices as followed by the farmers of the region was adopted. The crop was grown under irrigated conditions, and three irrigations were applied 21 days after sowing (DAS), 45 DAS, and 75 DAS. Nitrogen, phosphorus, and potassium at 120, 60, and 40 kg ha^−1^ were applied. All P and K and a half dose of N were applied at sowing as basal dose. The remaining half dose of N was top dressed in two equal splits at crown root initiation and boot stages. Hand weeding was carried out in the field to keep the field weed free. No infestation of insect pests was observed on the crop. Hence, no pesticide was applied during the period of the experiment. The occurrence of phenological events like tillering, booting, flowering, milk, dough, and physiological maturity in Julian Day was recorded from each plot, and average dates of these phases were calculated over the years and used for analysis. GDD, also known as heat unit (HU), was calculated at different phenological stages of the crop.

*Growing degree-day* is defined as the mean temperature above the base temperature. Mathematically, the GDD was computed by using the following equation (Nuttonson, 1955; Sastry and Chakravarty, 1982) at different phenological stages of the crop.


$$\text{GDD }=\;\Sigma \;\left[\left({\text{T}}_{\text{max}}+\;{\text{T}}_{\text{min}}\right)/2\;-\;{\text{T}}_{b}\right],$$

where GDD is the growing degree day (day °C), T_max_ is the daily maximum temperature (°C), T_min_ is the daily minimum temperature (°C), and T_b_ is the base temperature (°C); the base temperature was taken as 5°C (Nuttonson, 1955; Amrawat et al., 2013).

*Helio-thermal unit* was calculated by multiplying GDD and daily bright sunshine hours (BSS). BSS for a particular day as recorded by the sunshine recorder in the Agrometeorological Observatory was used in the estimation of HTU. The sum of HTU for the duration of each phenophase was calculated by using the following equation (Sastry and Chakravarty, 1982), and accumulated HTU at physiological maturity was calculated as follows:


HTU = GDD × n,

where n is the actual duration of bright sunshine hours.

Accumulated PTUs at physiological maturity were calculated by multiplying GDD with the length of maximum possible sunshine hours (Nuttonson, 1955; Sastry and Chakravarty, 1982). It is mathematically expressed as PTU = GDD × N, where N is the maximum possible sunshine hours.

Heat use efficiency (HUE), helio-thermal use efficiency (HTUE), and photo-thermal use efficiency (PTUE) were calculated following Singh and Khushu (2012).


$$\text{HUE }\left(\text{Kg h}{\text{a}}^{-1}{}^{\circ}\text{C da}y^{-1}\right)\;=\frac{{\text{Grain yield (Kg ha}}^{-1})}{\text{Accumulated GDD }({}^{\circ}\text{C day)}},$$

$$\text{PTUE }\left(\text{Kg h}{\text{a}}^{-1}{}^{\circ}\text{C day hou}{\text{r}}^{-1}\right)\;=\frac{{\text{Grain yield (Kg ha}}^{-1})}{\text{Accumulated PTU }({}^{\circ}\text{C day hour)}},$$

$$\text{HTUE }\left(\text{Kg h}{\text{a}}^{-1}{}^{\circ}\text{C day hou}{\text{r}}^{-1}\right)\;=\frac{\text{Grain yield }{\text{(Kg ha}}^{-1})}{\text{Accumulated HTU }({}^{\circ}\text{C day hour)}}.$$

Daily weather data on maximum and minimum temperatures for the growing season were collected from the nearby Agrometeorological Observatory, Dr. Rajendra Prasad Central Agricultural University, Pusa, Bihar.

Observations on yield attributes, viz., effective tillers per m^−2^, number of grains per spike, and test weight (1,000-grain), were taken from the net plot area. To determine the test weight of grains, 1,000 seeds from each plot were counted and dried until a constant weight was obtained. The crop was harvested manually with the help of a sickle from the net plot area. After the removal of excess moisture from grains of each plot, the grain yield and straw yield (kg per plot) were recorded after taking weight by open pan electronic balance, which was later converted to Mg ha^−1^. The harvest index (%) was calculated by dividing economic yield (grain) by the biological yield (grain + straw), as follows:


$$\text{Harvest Index }\left(\%\right)=\frac{\text{Economic yield }{\text{(Mg ha}}^{-1})}{\text{Biological yield }{\text{(Mg ha}}^{-1})}\;\times \;100.$$

Grain filling duration (GFD) was calculated by counting the number of calendar days from 50% flowering to physiological maturity. The grain filling rate (GFR) was calculated by dividing the grain yield (kg ha^−1^) by GFD (days).

Pearson’s correlation coefficients between grain yield, grain filling duration, and its rate, and yield attributes and weather parameters were computed (Gomez and Gomez, 1984). The sum of deviations of maximum and minimum temperatures from optimum thresholds was calculated at critical growth phases for each date of sowing, and based on the largest deviations, heat stress was identified for the crop sown on different sowing dates. In this study, the sum of deviation from optimum thresholds of maximum temperature and minimum temperature for the sensitive growth phases, viz., flowering to milking and flowering to maturity, was calculated by taking 25°C and 12°C as optimum threshold maximum and minimum temperature, respectively, for flowering to milking (F-Mlk) and 27°C and 14°C as threshold maximum and minimum temperatures for flowering to maturity (F-Mat) stages (Sattar et al., 2020 and Sattar and Srivastava, 2021).

The data on crop phenology, yield, and other parameters were subjected to appropriate statistical analysis through SPSS software (version 17.0), and the significance of mean values was compared using the least significant difference values (Gomez and Gomez, 1984).

Genotype-by-environment (GGE) biplot analysis was carried out using R software version 4.2.3 with the help of the GGE Biplots package to visualize and interpret the multi-environment data and performance of varieties (Saeidnia et al., 2023). Each sowing date was considered a test environment. Accordingly, five test environments were used for testing the varieties. Variety with higher yield and stability, and which-won-where pattern were visualized for studying the higher yield producing variety under different sowing environments.

We also used the CERES-Wheat model (DSSAT v. 4.75) to simulate wheat yields for the years 2050-51 and 2080-81 using the Intergovernmental Panel on Climate Change (IPCC)) AR5 scenario of Representative Concentration Pathway 4.5 (RCP 4.5). Soil data, crop management data, and weather data of the site were used as necessary inputs to run the CERES-Wheat model. For simulation purposes, the model required a set of genetic coefficients pertaining to the phenology and growth of wheat. The genetic coefficients of wheat cultivars, namely, RAU-3711, HD-2824, and HD-2733, were estimated by the genetic sub-model of the DSSAT with repeated interactions until a close match between simulated and observed parameters of phenology and yield was obtained (**Table 2**). With the use of RCP 4.5, the average projected yield based on these varieties for 2050-51 and 2080-81 is discussed in the article. The yield of wheat varieties grown during 2014–2015 was considered as a baseline for comparison with the projected yield.

**Table 2:** Genetic coefficient (GC) of wheat varieties.

S. no.	Genetic coefficients	GC of wheat varieties		
		RAU-3711	HD 2824	HD 2733
1.	PIV: days at optimum vernalizing temperature required to complete vernalization	14	17	18
2.	PID: percentage reduction in development rate in a photoperiod 10 h shorter than the threshold relative to the threshold	39	42	44
3.	P5: grain filling (excluding lag) phase duration	535	495	520
4.	PHINT: interval between successive leaf tip appearances	95	95	95
5.	G1: kernel number per unit canopy weight at anthesis (mg/g)	20	22	17
6.	G2: standard kernel size under optimum conditions (mg)	36	37	34
7.	G3: standard non-stressed dry weight (total including grain) of a single tiller at maturity (g)	1.5	1.5	1.5

## 3 Results and discussion

### 3.1 Wheat phenology and grain filling duration under varying sowing environments

The phenological development of a crop is the most important biological footprint of climate change and warming impact. The study of crop phenology has important implications to understand crop response and adaptation to climate change (Tao et al., 2022). In the present study, crop micro-environment as imposed by different dates of sowing significantly influenced thermal days required to achieve different phonological stages, viz., tiller initiation, booting, 50% flowering, milking, dough, and maturity (**Tables 3**, **4**). It extended from 26 to 38, 61 to 79, 82 to 97, 81 to 104, 99 to 132, and 106 to 139 days to reach tiller initiation, booting, 50% flowering, milking, dough, and maturity, respectively, over the experimentation period (2014–2015 to 2016–2017). Many authors (Tao et al., 2012; Li et al., 2021; Tao et al., 2022) opined that the phenology of crops is intricately related to crop management, sowing dates, and cultivars. In the Indo-Gangetic Plains, forced maturity of wheat due to a sudden rise in ambient temperature is common during the post-heading phase of the crop (Ram et al., 2012). Hence, quantification of the exact duration of phenological stages in a particular crop-growing environment and their impact on the yield is very important (Amrawat et al., 2013). This is more pertinent concerning the effect of changing climate on crop phenology (Liu et al., 2018). In the present study, the crop planted on 15 November took 78, 91, 100, 130, and 138 days to achieve booting and 50% flowering, milking, dough, and maturity stages, respectively, which were significantly higher than the rest of the sowing dates. However, the highest days to reach tiller initiation (37) were associated with the crop planted on 25 December (D5), which was significantly higher than the rest of the sowing dates. Days to tiller initiation varied from 27 days in D2 to 37 days in D5 (**Table 2**). In general, with progress in the date of sowing, tiller initiation was delayed. However, a reverse trend was recorded for days before booting. Tillering stage of the crop sown on later dates encountered lower temperatures. There was a difference of 16 days to complete booting for D1 and D5. Similarly, days to 50% flowering showed a declining trend as that of the booting stage, and D1 took 91 days to attain 50% flowering, which was significantly higher than that of all other dates of sowing. There was a difference of 18 days to reach 50% flowering for D1 and D5. A similar trend was also observed for the milking, dough, and maturity stages. There was a difference of 18, 29, and 30 calendar days to reach the milking, dough, and maturity stages, respectively, between D1 and D5. D1 took 138 days to reach the maturity stage, while all other dates took significantly lower days to reach the maturity stage. The 3-year average data revealed that the duration of different phenophases differed significantly due to cultivars except for tiller initiation (**Tables 3**, **4**). Systematic and accurate records of crop phenological data along with information on cultivars and management practices allow researchers to study the effects of weather on crop productivity based on actual weather data (Tao et al., 2012; Palosuo et al., 2015; Tao et al., 2017). This helps in better understanding the impact of adaptation mechanisms and studying the impact of future climate on crop production. Sattar et al. (2020) reported a reduced duration of crop phenology in response to elevated temperature. Late-sown crops had to encounter higher temperatures during critical phenophases. Out of three cultivars, HD-2733 and HD-2824 registered a similar number of days to complete different phenophases. In contrast, RAU-3711 availed significantly lesser days to attain booting (69), 50% flowering (79), milking (88), dough (114), and maturity (114) stages when compared to HD-2824 and HD-2733.

**Table 3:** Phenology of wheat (thermal days) as affected by sowing environment and cultivars.

Date of sowing (D)	Tiller initiation				Booting				50% Flowering			
	2014–15	2015–16	2016–17	Average	2014–15	2015–16	2016–17	Average	2014–15	2015–16	2016–17	Average
D1	31b	26c	27c	28cd	79a	79a	75a	78a	90a	97a	86a	91a
D2	30b	26c	26c	27d	74b	76b	73b	74b	86b	89b	83b	86b
D3	31b	27bc	30b	29c	69c	69c	73b	70c	78c	79c	82b	80c
D4	37a	28b	31b	32b	66d	65d	66c	66d	76c	74d	79c	76d
D5	38a	35a	36a	37a	61e	65d	61d	62e	72d	75d	73d	73e
Cultivars (C)												
RAU-3711	33	28	30	30ns	67b	70b	69b	69b	77b	82b	79b	79b
HD-2824	34	28	30	31ns	71a	71ab	69b	71a	82a	83a	81a	82a
HD-2733	34	28	30	31ns	71a	72a	70a	71a	81a	83a	82a	82a
D × C	NS	NS	NS	NS	NS	NS	NS	NS	NS	NS	S	NS

Values with at least a common letter down the column are not significantly different from each other according to LSD test (p< 0.05).

LSD, least significant difference; S, significant; NS, non-significant; D1, 15 November; D2, 25 November; D3, 5 December; D4, 15 December; D5, 25 December.

**Table 4:** Phenology of wheat (thermal days) as affected by sowing environment and cultivars.

Date of sowing (D)	Milking				Dough				Physiological maturity			
	2014–15	2015–16	2016–17	Average	2014–15	2015–16	2016–17	Average	2014–15	2015–16	2016–17	Average
D1	100a	104a	96a	100a	128a	132a	131a	130a	135a	139a	139a	138a
D2	94b	95b	91b	93b	120b	122b	123b	122b	127b	129b	130b	129b
D3	85c	88c	91b	88c	111c	114c	118c	115c	118c	121e	123c	121c
D4	85c	84d	88c	86d	106d	106d	112d	108d	113d	113d	118d	115d
D5	81d	83d	81d	82e	99e	100e	103e	101e	106e	107e	110e	108e
Cultivars (C)												
RAU-3711	86b	91	88b	88b	110b	116a	117	114b	118	123a	123	121c
HD-2824	91a	91	90a	90a	114a	114b	117	115ab	120a	121b	124	122b
HD-2733	90a	91	91a	90a	114a	115ab	118	116a	121a	122ab	125	123a
D × C	NS	NS	NS	NS	NS	NS	NS	NS	NS	S	NS	NS

Values with at least a common letter down the column are not significantly different from each other according to LSD test (p< 0.05).

LSD, least significant difference; S, significant; NS, non-significant; D1, 15 November; D2, 25 November; D3, 5 December; D4, 15 December; D5, 25 December.

Grain filling duration and grain filling rate of wheat were significantly affected during 3 years of experimentation (**Table 5**). GFD, in general, showed a declining trend with the advancement of sowing dates. D1 had a grain filling duration of 47 days, which was significantly higher than all other dates of sowing. The minimum grain filling duration (34 days) was associated with D5. Delayed sowing beyond D3 significantly reduced the GFD across varieties except for RAU-3711, which gave statistically similar GFD when compared with the highest GFD for 2015–2016. Kheiri et al. (2021) reported that variations in the length of the grain filling period contributed to significant changes in the grain yield of wheat cultivars *Sardari* and *Azar2* in Iran. A 5°C increase in temperature above 20°C increased the rate of grain filling and shortened the grain filling duration by 12 days in wheat (Yin et al., 2009). In this study, the highest GFR for 2014–2015, 2015–2016, and 2016–2017 was recorded with D2 (111 kg ha^−1^ day^−1^), D3 (55 kg ha^−1^ day^−1^), and D3 (63 kg ha^−1^ day^−1^), respectively. The pooled data indicated that the grain filling rate increased up to D3 and then started declining up to D5, implying that wheat yield decreased sharply beyond D3. This might be due to increased thermal load on the crop, which is manifested in terms of higher accumulation of GDD due to delayed sowing. Chen et al. (2018) reported a shortening of the reproductive period due to an increase in GDD and extreme temperature (34°C) degree days (EDD). The highest GFD was recorded with the cultivar RAU-3711 (**Table 5**), whereas HD-2824 recorded the highest GFR. Exposing the crop to higher temperatures at critical growth phases tends to significantly affect phenophase duration and crop yield (Parya et al., 2010). Poudel et al. (2021) reported that the optimum temperature during the anthesis and grain filling stage ranges from 12°C to 22°C. High temperatures greater than 22°C during anthesis to grain maturity reduced grain yield due to a decrease in grain filling duration (Joshi et al., 2007). Shortening of grain filling duration is a serious problem in wheat owing to higher average temperature during the post-heading period (Lobell et al., 2012; Garg et al., 2013).

**Table 5:** Effect of sowing environment and cultivars on grain filling duration and rate.

Date of sowing (D)	Grain filling duration (days)				Grain filling rate (kg ha^−1^ day^−1^)			
	2014–15	2015–16	2016–17	Average	2014–15	2015–16	2016–17	Average
D1	45a	42a	53a	47a	90b	53a	53b	66bc
D2	41b	40b	47b	43b	111a	54a	61a	75a
D3	41b	42a	41c	41b	105a	55a	63a	74a
D4	37c	39b	39d	38c	104a	47a	59a	70ab
D5	33d	33c	37e	34d	105a	36b	49b	64c
Cultivars (C)								
RAU-3711	40ns	40a	44ns	42a	94b	50ab	58ns	67b
HD-2824	38ns	39b	43ns	40b	106a	53a	56ns	72a
HD-2733	40ns	39b	42ns	40b	109a	45b	58ns	71ab
D × C	NS	S	NS	NS	NS	NS	S	S

Values with at least a common letter down the column are not significantly different from each other according to LSD test (p< 0.05).

LSD, least significant difference; S, significant; NS, non-significant; D1, 15 November; D2, 25 November; D3, 5 December; D4, 15 December; D5, 25 December.

### 3.2 Yield attributes, yield, and harvest index under varying sowing environments

**Figure 1:**
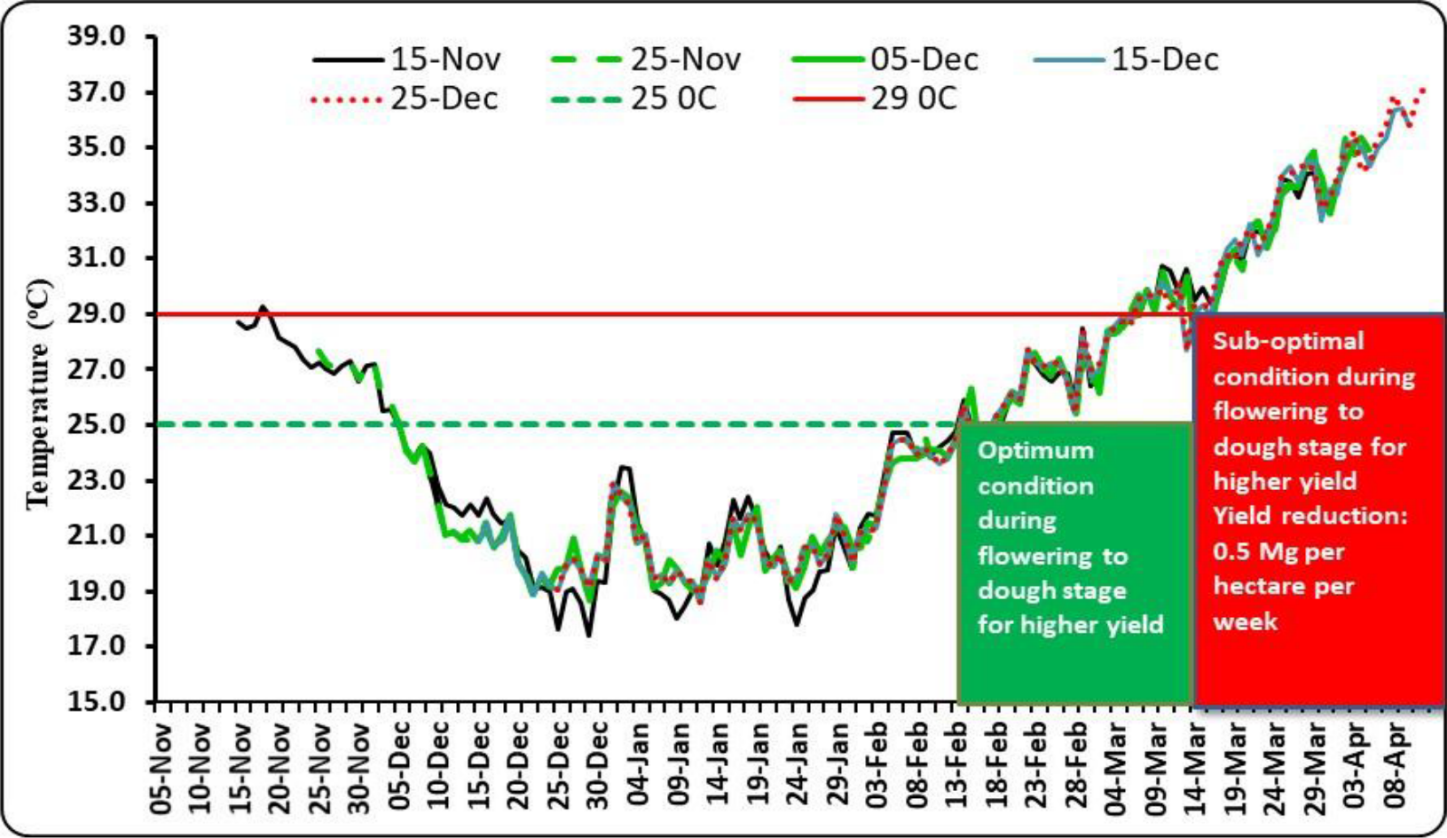
Optimal and sub-optimal conditions during flowering to grain filling stage for higher yield of wheat.

**Figure 2:**
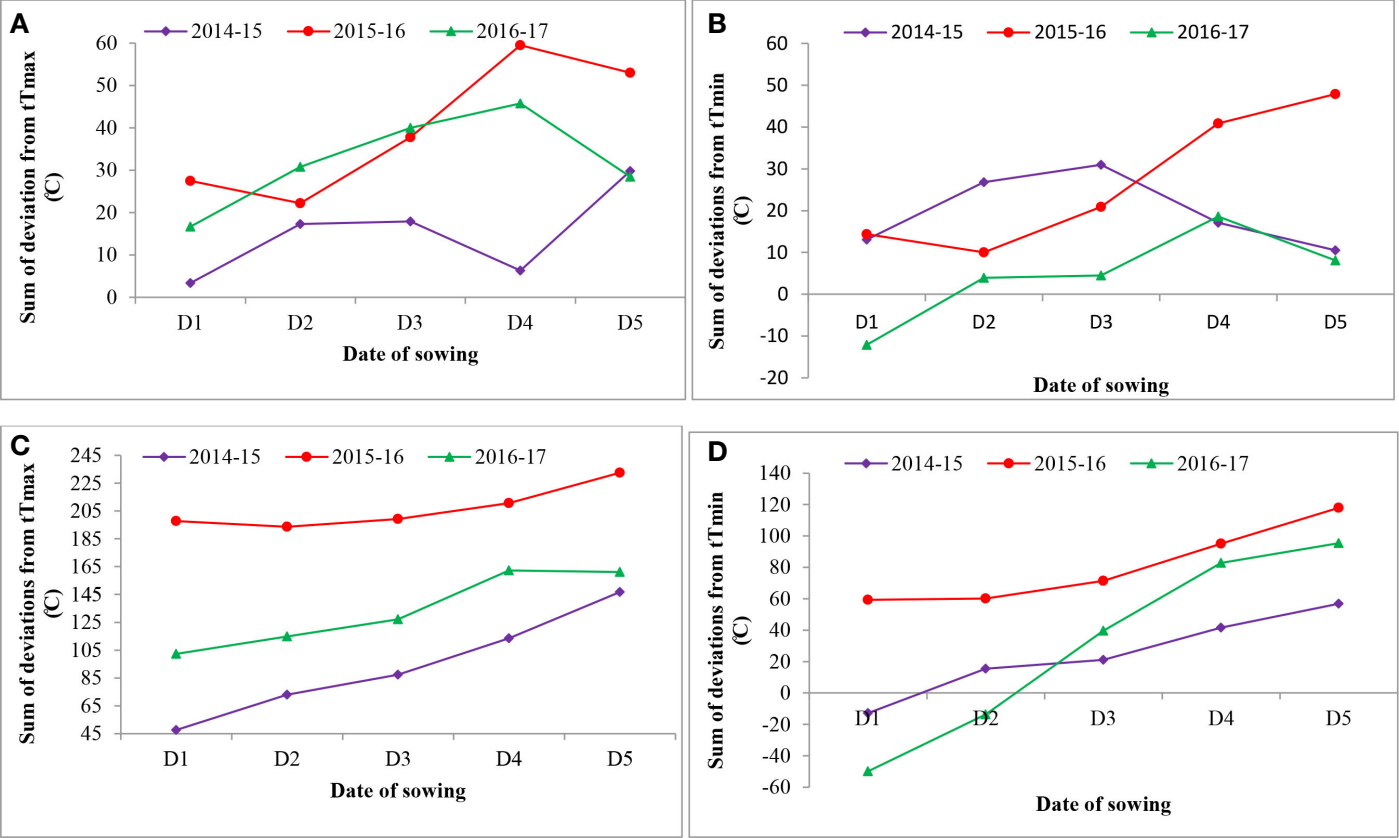
Effect of sowing environment on sum of deviations from threshold maximum and minimum temperature from flowering to milking stage **(A, B)** and flowering to maturity stage **(C, D)** in wheat. tTmax, threshold maximum temperature; tTmin, threshold minimum temperature.

The date of sowing produced a significant effect on the number of effective tillers per m^2^. D2 generated the highest number of effective tillers among all the dates of sowing (**Table 6**). The reduced number of tillers for the crop sown after 25 November might be due to the survival of less number of tillers under high-temperature conditions. Temperature decides the tiller initiation process in the axils of the basal leaves of wheat plants, and under unfavorable thermal regimes, the process of development of tillers either slows down or stops (Rahman et al., 2009). In our study, the impact of heat stress on the number of effective tillers was clearly visible. During 2014–2015, which did not experience any heat stress, effective tillers per m^2^ were observed to be the maximum (489.3), which was significantly higher as compared to that of the other two seasons. However, under the unfavorable thermal regime, the effective tillers per m^2^ reduced to 285.6 during 2015–2016 (**Supplementary Table 1**). The highest number of grains per spike was recorded with D3 for average data (**Table 6**). However, due to variations in temperature regime during the post-heading to grain filling period in different growing seasons, the maximum number of grains per spike was found to differ substantially from year to year. Considering test weight, it showed a declining trend with the advancement of sowing date, perhaps due to increased thermal load on the crop, causing grain shrinkage under the production of reactive oxygen species, reduced pollen tube development, increased pollen mortality, and grain abortion (Nawaz et al., 2013; Dubey et al., 2019). Among the years, the crop during 2014–2015 recorded the highest test weight (40.7 g), which was significantly higher than the other 2 years. The lowest test weight (30.4) was noted during 2015–2016, wherein the crop faced severe heat stress (**Supplementary Table 1**). In the case of cultivars, the highest number of effective tillers per m^2^ was associated with HD-2733 for all the years of experimentation, showing thermo-tolerant characteristics, and thus, this cultivar resisted the negative impact of seasonal temperature increase on active tillering. Similarly, cultivars caused significant variation in the number of grains per spike except for 2014. Poudel et al. (2020) recorded heat stress-induced reduction in tiller number and spikelets per spike in wheat, resulting in poor grain yield. Higher day length reduced the spikelet number by decreasing the initiation period (Masoni et al., 2001; Arduni et al., 2009). Owing to the differential thermal regime experienced by wheat planted under different sowing windows, grain yield was found to be significantly affected (**Table 7**). Grain yield increased from D1 to D2/D3 and then declined down to D5 over 3 years of experimentation. For a higher yield of wheat (>4.0 Mg ha^−1^) in the region, it is necessary that the period from flowering to dough stage must be completed by 15 March, beyond which yield decreases significantly (0.5 Mg ha^−1^ per week) due to high-temperature stress. The optimal and sub-optimal conditions for wheat growth based on yields, normal weather of congenial, and a heat stress year are given in **Figure 1**. The maximum grain yield was achieved with D2 for 2014–2015 and 2016–2017 (4.55 and 2.87 Mg ha^−1^, respectively), while D3 gave the highest grain yield for 2015–2016 (2.31 Mg ha^−1^). Average data of 3 years showed that D2 produced the highest grain yield (3.21 Mg ha^−1^), 48.61% higher than D5 (2.16 Mg ha^−1^). Grain yield obtained with D2 and D3 were statistically similar. Among the years, the highest grain yield was observed in 2014–2015 (4.03 Mg ha^−1^), which was significantly higher than the other 2 years (**Supplementary Table 1**). However, the lowest grain yield was observed in 2015–2016 (1.96 Mg ha^−1^). The crop during this year experienced the highest heat stress (**Figure 2**). Higher grain yield during 2014–2015 compared to the rest of the years could be ascribed to a congenial thermal regime that favored the production of higher effective tillers per m^2^ (489.3) and test weight (40.7 g). Dubey et al. (2019) reported yield loss of wheat at New Delhi by 70, 29, and 77 kg ha^−1^ per day due to delay in sowing beyond the first week of November in varieties, viz., HD-2932, WR-544 and HD-2967 respectively. In one study conducted by Poudel et al. (2021), the grain yield of wheat was reduced by 47.6% under heat-stress conditions.

**Table 6:** Yield attributes of wheat as affected by sowing environment and cultivars.

Date of sowing (D)	Effective tillers per m^2^				No. of grains per spike				Test weight (g)			
	2014–15	2015–16	2016–17	Average	2014–15	2015–16	2016–17	Average	2014–15	2015–16	2016–17	Average
D1	431b	330	337a	366a	46a	44	52	47	46.6a	32.0a	33.1a	37.2a
D2	513a	291	298ab	367a	45a	41	49	45	44.4a	32.9a	34.0a	37.1a
D3	474ab	293	290bc	352ab	44ab	46	53	48	41.2b	31.3a	32.3a	34.9a
D4	453ab	239	250c	314b	40bc	45	53	46	38.6b	28.8ab	29.9ab	32.4b
D5	426b	276	284bc	329ab	38c	44	52	45	32.8c	26.9b	27.6b	29.1b
Cultivars (C)												
RAU-3711	411b	286	294ab	331b	45	49a	57a	50a	42.4a	28.8	29.7	33.6
HD-2824	476a	265	267b	336b	42	43b	50b	45b	39.3b	31.6	32.6	34.5
HD-2733	491a	306	313a	370a	42	40b	48b	43b	40.4ab	30.8	31.9	34.4
D × C	NS	NS	S	NS	NS	NS	NS	NS	S	NS	NS	NS

Values with at least a common letter down the column are not significantly different from each other according to LSD test (p< 0.05).

LSD, least significant difference; S, significant; NS, non-significant; D1, 15 November; D2, 25 November; D3, 5 December; D4, 15 December; D5, 25 December.

**Table 7:** Grain yield of wheat as affected by sowing environment and cultivars.

Date of sowing (D)	Grain yield (Mg ha^−1^)				Straw yield (Mg ha^−1^)				Biological yield (Mg ha^−1^)				Harvest index (%)			
	2014–15	2015–16	2016–17	Average	2014–15	2015–16	2016–17	Average	2014–15	2015–16	2016–17	Average	2014–15	2015–16	2016–17	Average
D1	4.06b	2.24a	2.78b	3.03b	6.16a	4.49ab	5.19a	5.28a	10.22bc	6.73a	7.59ab	8.18a	39.96bc	33.21a	38.37	37.18ab
D2	4.55a	2.20a	2.87a	3.21a	6.45a	4.58ab	5.35a	5.46a	10.99a	6.78a	7.70a	8.49a	41.36a	32.46a	39.09	37.64a
D3	4.25ab	2.31a	2.57c	3.04ab	6.19a	4.91a	5.27a	5.46a	10.44ab	7.21a	7.74a	8.46a	40.62ab	32.14a	33.99	35.58bc
D4	3.80c	1.86b	2.30d	2.65c	5.77b	3.92bc	4.64a	4.78b	9.56cd	5.77b	6.63b	7.32b	39.69bc	32.33a	35.08	35.70b
D5	3.49c	1.18c	1.82e	2.16d	5.51b	3.38bc	3.70b	4.20c	9.00d	4.55c	4.96c	6.17c	38.62c	25.83b	36.84	33.76c
Cultivars (C)																
RAU-3711	3.77b	2.04a	2.53a	2.78	5.83b	4.52	5.14	5.16	9.60b	6.56a	7.33a	7.83	39.13b	30.55	36.28	35.32
HD-2824	4.02ab	2.09a	2.42b	2.84	5.85b	4.30	4.82	4.99	9.87b	6.38ab	7.05ab	7.77	40.66a	32.52	35.19	36.12
HD-2733	4.30a	1.74b	2.46b	2.83	6.36a	3.96	4.52	4.95	10.66a	5.69b	6.39b	7.58	40.35a	30.51	38.57	36.48
D × C	NS	S	S	S	S	NS	NS	S	S	S	S	S	S	NS	S	S

Values with at least a common letter down the column are not significantly different from each other according to LSD test (p< 0.05).

LSD, least significant difference; S, significant; NS, non-significant; D1, 15 November; D2, 25 November; D3, 5 December; D4, 15 December; D5, 25 December.

**Figure 3:**
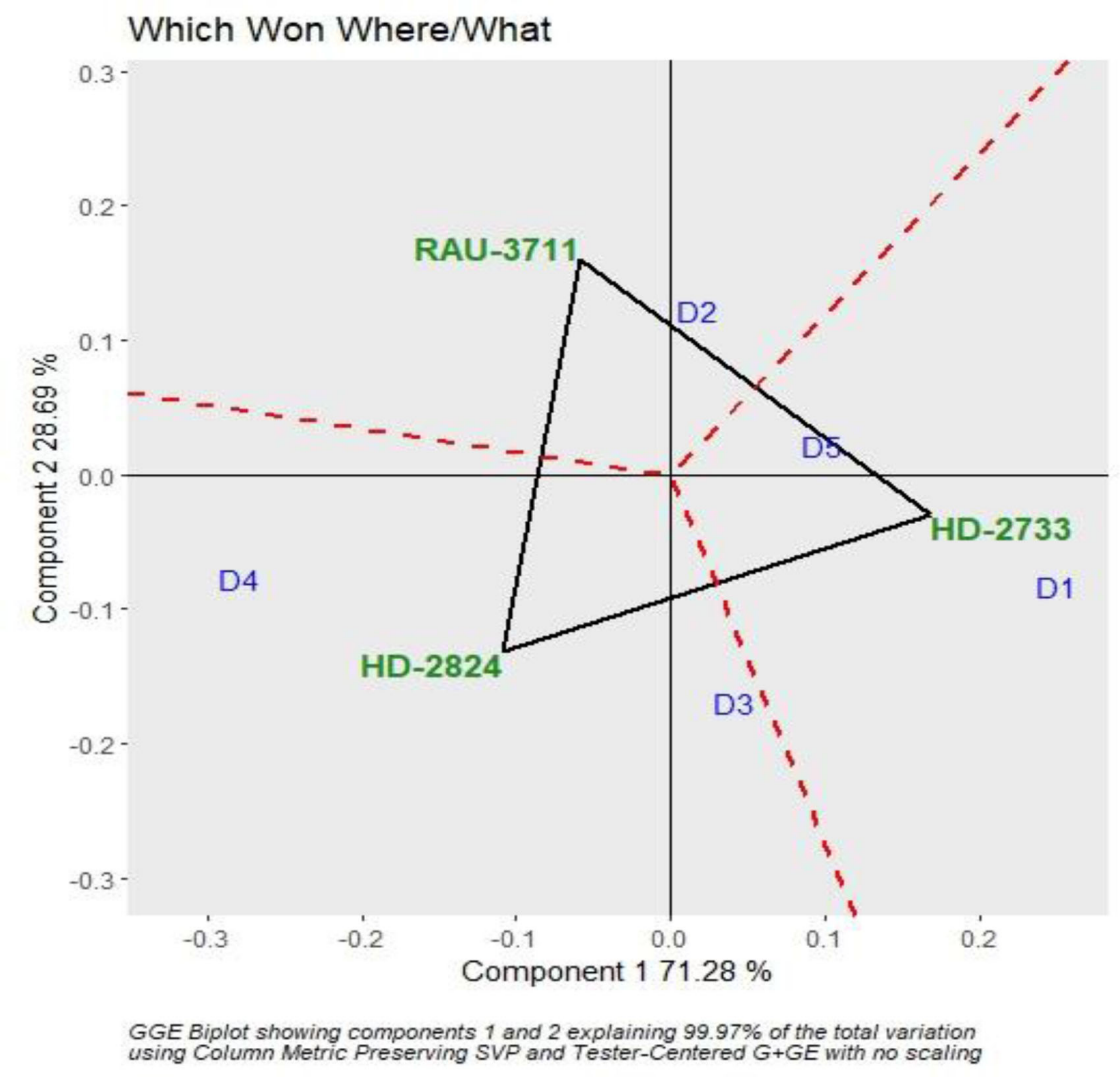
Polygon view of GGE biplot depicting varietal performance under various sowing dates. GGE, genotype by environment.

**Figure 4:**
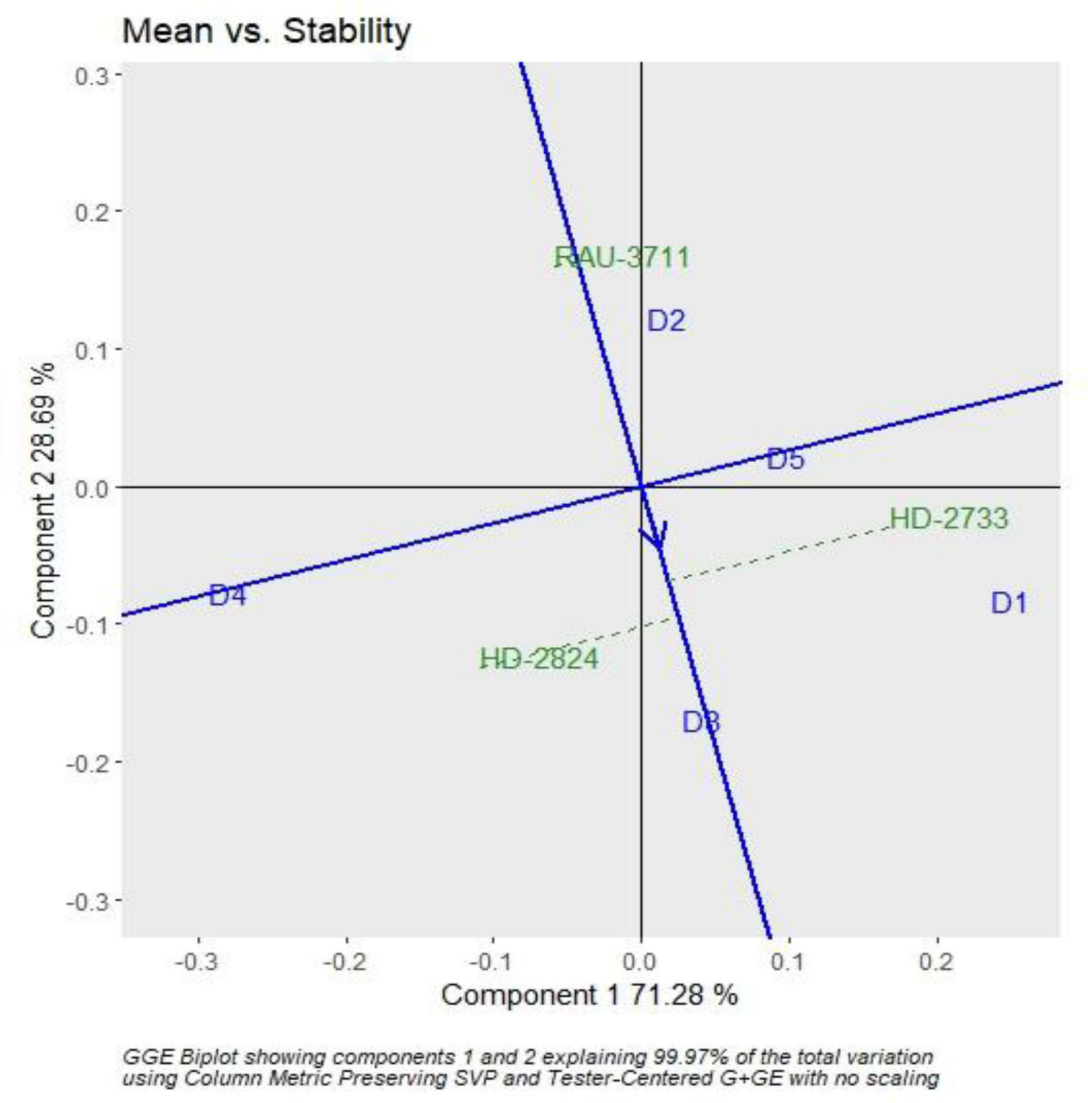
GGE biplot depicting the ranking of wheat varieties based on grain yield performance and stability. GGE, genotype by environment.

The interaction effect of sowing date and varieties were found significant for grain yield. Hence, genotype plus GGE biplot analysis shows the magnitude and pattern of the genotype–environment interaction effect among the genotypes in a graphical way. It revealed that the first two principal components (PCs) accounted for 70.37% and 28.56% variation of genotype + genotype–environment sum of squares, explaining a total of 98.93% variation (**Figures 3**, **4**). The which-won-where pattern of the interaction between the date of sowing and varieties for grain yield (**Figure 3**) showed that variety RAU-3711 produced the highest grain yield when sown on 25 November (D2). Similarly, HD-2824 produced the highest grain yield when sown on 5 December (D3) and 15 December (D4). However, HD-2733 produced the highest grain yield when sown on 15 November (D1) and 25 December (D5). The average grain yield and stability performance of varieties are graphically depicted through the average environment coordination method (**Figure 4**), which helped in identifying the highest-yielding and most stable variety. The single-arrowed line that passes through the origin of the biplot and points toward higher mean values is the AEC abscissa, whereas the other line in the graph depicts the AEC ordinate. The variety farthest from the origin on the positive side of the AEC abscissa has the highest grain yield, and that farthest from the origin on the negative side of the AEC abscissa has the lowest grain yield. Therefore, the variety HD-2824 recorded the highest grain yield followed by HD-2733 and RAU-3711. However, the greater the absolute length of the projection of a variety, the less stable it is. As per **Figure 4**, the variety RAU-3711 was the most stable among the three varieties.

Straw yield followed a similar trend as grain yield for yearly data, but average data showed both D2 and D3 recorded the same straw yield (5.46 Mg ha^−1^), which was 30% higher than that of the crop planted on D5 (**Table 7**). Biological yield (BY) increased from D1 to D2/D3 and then declined to D5 over 3 years of experimentation. It followed a similar pattern as that of grain yield, perhaps due to the partitioning of photosynthates synchronized linearly with temperature variation. The highest BY for 2014, 2015, and 2016 and average data was recorded with D2, D3, D3, and D2, respectively. Average data showed that BY increased from D1 to D2 and then started declining. The highest BY was recorded with D2 (8.49 Mg ha^−1^), which was comparable with that of D3 (8.46 Mg ha^−1^) and D1 (8.18 Mg ha^−1^). D2 recorded 37.60% higher BY than D5. Delayed sowing shortens the crop growth duration and, consequently, the amount of radiation interception by the crop canopy. In response to this, biomass and yield tend to decrease significantly for the late-sown crop (Tao et al., 2015). The date of sowing significantly influenced the harvest index during 2014 and 2015 and for average data. For 2014–2015 and average data, it increased till D2, after which it started declining. However, for 2015–2016, it declined with the advancement of sowing dates. The highest harvest index for 2014–2015, 2015–2016, 2016–2017, and average data was recorded with D2, D1, D2, and D2, respectively. Varieties caused significant yield variation during 3 years of experimentation (**Table 7**). The highest grain yield for 2014–2015, 2015–2016, and 2016–2017 were obtained with HD-2733 (4.30 Mg ha^−1^), HD-2824 (2.09 Mg ha^−1^), and RAU-3711 (2.53 Mg ha^−1^), respectively. Similarly, the straw yield was significantly affected only during 2014–2015, where HD-2733 recorded a significantly higher straw yield (6.36 Mg ha^−1^) than the other two varieties. The average data showed that the effect of cultivar was non-significant for straw yield. The effect of varieties on BY was significant for 2014–2015, 2015–2016, and 2016–2017. However, for average data, it was non-significant. The highest BY for 2014–2015, 2015–2016, and 2016–2017 was observed with HD-2733, RAU-3711, and RAU-3711, respectively.

Varieties had a non-significant effect on harvest index (HI) except for the years 2014–2015 (**Table 7**). The highest HI for 2014–2015 was recorded with HD-2824 (40.66), which was comparable with HD-2733 (40.35). Similarly, HD-2824, HD-2733, and HD-2733 recorded the highest HI for 2015–2016, 2016–2017, and average data. When the sowing was delayed beyond 25 November, late-sown crops had to experience higher temperatures during the period of experimentation. HI decreased for later sown crops due to exposure to high temperatures. Dubey et al. (2019) linked the reduction of HI under the late sown condition of wheat with the greenness index and grain filling period under heat stress. High temperature during the grain filling period shrinks the size of grains in wheat apart from reduced grain filling duration (Asseng et al., 2011; Lobell et al., 2012; Tao et al., 2015). In wheat, the period from the onset of spike initiation to flowering is very sensitive to temperature acceleration, and it seems to be the main reason for the reduction in sink size under high-temperature conditions, resulting in poor grain yield (Poudel et al., 2021). However, a normal sowing window provides an opportunity to accumulate more biomass as compared to late sowing due to a longer growing period, which helps produce a higher grain yield (Singh and Pal, 2003; Dar Eajaz et al., 2018).

### 3.3 Influence of sowing environment on the accumulation of agrometeorological indices

Air temperature modifies the enzymatic functions of plants and causes a change in phenology, which is directly related to yield (Zhu et al., 2018). The effect of temperature on crops can be effectively explained through the GDD concept. Hence, agrometeorological indices, viz., GDDs and photo-thermal index, have great practical significance in evaluating phenology and growth parameters (Streck et al., 2008; Kumar et al., 2010). Sub-optimal photo-thermal regimes during crop growing season have a profound impact on crop yield. Considering heat unit accumulation by wheat sown on different dates, it was observed that accumulated HU varied at 264°C–445°C, 640°C–917°C, 804°C–1,156°C, 926°C–1,269°C, 1,309°C–1,808°C, and 1,456°C–1,960°C days for tiller initiation, booting, 50% flowering, milking, dough, and maturity, respectively, over the experimentation period (**Tables 8**, **9**). The average data showed that the D1 accumulated 408°C, 894°C, 1,056°C, 1,190°C, 1,709°C, and 1,860°C days for completion of tiller initiation, booting, 50% flowering, milking, dough, and maturity stages of wheat, respectively, which were significantly higher than rest of the sowing dates. Similarly, accumulated PTUs and HTU at physiological maturity were found to vary significantly with sowing environments and cultivars (**Table 10**). In order to harness the maximum benefits of the ambient thermal environment for higher yield, it is vital that sowing is completed at the right time. Inappropriate sowing dates cause varied weather conditions, especially in terms of the thermal requirement and radiation received by the crop canopy. In this context, an agrometeorological index such as GDD integrates phenological behavior with the thermal regime since it has a direct relation with the growth and development of crops (Mishra et al., 2007).

**Table 8:** Growing degree day (heat unit) (°C days) at different phenophases of wheat as influenced by sowing environments and cultivars.

Date of sowing (D)	Tiller initiation stage				Booting stage				50% Flowering stage			
	2014–15	2015–16	2016–17	Average	2014–15	2015–16	2016–17	Average	2014–15	2015–16	2016–17	Average
D1	445a	400a	380b	408a	855a	917a	910a	894a	975a	1,156a	1,038a	1,056a
D2	351b	335b	325d	337c	761b	840b	841b	814b	906b	1,031b	961b	966b
D3	315d	281c	334cd	310d	696c	718d	807c	740c	804c	885d	956b	882c
D4	331cd	264d	339c	312d	657d	684e	752d	698d	826c	824e	963b	871c
D5	350bc	341b	394a	362b	640e	755c	712e	702d	817c	933c	912c	887c
Cultivars (C)												
RAU-3711	356	324	351	344	687c	769b	793c	750b	823b	958b	941c	907b
HD-2824	367	324	359	350	747a	786a	803b	779a	893a	964ab	969b	942a
HD-2733	353	325	353	344	732b	793a	817a	781a	880a	975a	989a	948a
D × C	NS	NS	NS	NS	NS	NS	NS	NS	NS	NS	S	NS

Values with at least a common letter down the column are not significantly different from each other according to LSD test (p< 0.05).

LSD, least significant difference; S, significant; NS, non-significant; D1, 15 November; D2, 25 November; D3, 5 December; D4, 15 December; D5, 25 December.

**Table 9:** Growing degree day (heat unit) (°C days) at different phenophases of wheat as influenced by sowing environments and cultivars.

Date of sowing (D)	Milking stage				Dough stage				Physiological maturity stage			
	2014–15	2015–16	2016–17	Average	2014–15	2015–16	2016–17	Average	2014–15	2015–16	2016–17	Average
D1	1,128a	1,269a	1,172a	1,190a	1,574a	1,808a	1,746a	1,709a	1,730a	1,960a	1,890a	1,860a
D2	1,041b	1,132b	1,088b	1,087b	1,461b	1,651b	1,619b	1,577b	1,613b	1,805b	1,769b	1,729b
D3	926d	1,027d	1,086b	1,013c	1,362c	1,538c	1,576e	1,492c	1,512c	1,688c	1,692c	1,630c
D4	954c	1,008e	1,106b	1,022c	1,340c	1,443d	1,558e	1,447d	1,487cd	1,617d	1,707c	1,604d
D5	953c	1,086c	1,029c	1,023c	1,309d	1,451d	1,465d	1,408e	1,456d	1,617d	1,623d	1,565e
Cultivars (C)												
RAU-3711	954c	1,101b	1,076b	1,044b	1,354b	1,590a	1,587	1,510b	1,515b	1,750a	1,727	1,664b
HD-2824	1,033a	1,103a	1,098a	1,078a	1,435a	1,565b	1,590	1,530a	1,577a	1,725b	1,736	1,679ab
HD-2733	1,015b	1,110a	1,114a	1,080a	1,438a	1,579ab	1,601	1,539a	1,587a	1,737ab	1,746	1,690a
D × C	NS	NS	NS	NS	NS	NS	NS	NS	NS	NS	NS	NS

Values with at least a common letter down the column are not significantly different from each other according to LSD test (p< 0.05).

LSD, least significant difference; S, significant; NS, non-significant; D1, 15 November; D2, 25 November; D3, 5 December; D4, 15 December; D5, 25 December.

**Table 10:** Effect of sowing environment and cultivars on accumulated PTU and HTU by wheat.

Date of sowing (D)	Accumulated PTU (°C day hour)				Accumulated HTU (°C day hour)			
	2014–15	2015–16	2016–17	Average	2014–15	2015–16	2016–17	Average
D1	19,313a	22,132a	21,377	20,941a	7,809a	10,158a	10,263a	9,410a
D2	18,208b	20,430b	19,976	19,538b	7,207b	9,857b	9,421b	8,828c
D3	17,157c	19,222c	17,525	17,968c	7,147b	9,575c	9,602b	8,775c
D4	17,109c	18,434e	19,610	18,384bc	7,625a	9,642c	10,372a	9,213b
D5	16,848c	18,727d	18,792	18,122c	7,763a	10,014ab	10,268a	9,348a
Cultivars (C)								
RAU-3711	17,191b	19,977a	18,632	18,600	7,218b	9,949a	9,919	9,029b
HD-2824	17,911a	19,585b	19,774	19,090	7,610a	9,746b	9,974	9,110ab
HD-2733	18,079a	19,806a	19,962	19,282	7,703a	9,853ab	10,061	9,206a
D × C	NS	S	NS	NS	NS	NS	NS	NS

Values with at least a common letter down the column are not significantly different from each other according to LSD test (p< 0.05).

LSD, least significant difference; PTU, photo-thermal unit; HTU, helio-thermal unit; D1, 15 November; D2, 25 November; D3, 5 December; D4, 15 December; D5, 25 December.

The date of sowing significantly affected HUE during all the years of experimentation (**Table 11**). HUE increased up to D2 and thereafter declined until D5 during 2014–2015 and 2016–2017, but for 2015–2016 and average data, it increased up to D3 and thereafter declined until D5. PTUE and HTUE were observed to be higher for the crop planted from 25 November to 5 December. Progressive delay in sowing corresponded to an increase in temperature, causing a shortening of the crop growing period leading to lower yield and higher accumulated HU. Dar Eajaz et al. (2018) linked lower thermal use efficiency of delayed sown wheat beyond the optimum window to lower yield under high moisture stress.

**Table 11:** Effect of sowing environment and cultivars on HUE, PTUE, and HTUE of wheat.

Date of sowing (D)	HUE (kg/°C days)				PTUE (kg/°C day hour)				HTUE (kg/°C day hour)			
	2014–15	2015–16	2016–17	Average	2014–15	2015–16	2016–17	Average	2014–15	2015–16	2016–17	Average
D1	2.34b	1.15b	1.47b	1.65b	0.21b	0.10a	0.13	0.15	0.52b	0.22ab	0.27b	0.34b
D2	2.82a	1.22ab	1.62a	1.89a	0.25a	0.11a	0.14	0.17	0.63a	0.22ab	0.30a	0.39a
D3	2.81a	1.37a	1.52b	1.90a	0.25a	0.12a	0.30	0.22	0.59a	0.24a	0.27b	0.37a
D4	2.55b	1.15b	1.35c	1.68b	0.22b	0.10a	0.12	0.15	0.50bc	0.19b	0.22c	0.30c
D5	2.39b	0.73c	1.12d	1.42c	0.21b	0.06b	0.10	0.12	0.45c	0.12c	0.18d	0.25d
Cultivars (C)												
RAU-3711	2.49b	1.16a	1.46a	1.71	0.22	0.10ab	0.23	0.18	0.53	0.21a	0.26a	0.33
HD-2824	2.56ab	1.20a	1.39b	1.72	0.22	0.11a	0.12	0.15	0.53	0.21a	0.24b	0.33
HD-2733	2.71a	1.00b	1.40b	1.70	0.24	0.09b	0.12	0.15	0.56	0.18b	0.25ab	0.33
D × C	NS	S	S	S	NS	S	NS	NS	NS	S	S	S

Values with at least a common letter down the column are not significantly different from each other according to LSD test (p< 0.05).

LSD, least significant difference; HUE, heat use efficiency; PTUE, photo-thermal use efficiency; HTUE, helio-thermal use efficiency; S, significant; NS, non-significant; D1, 15 November; D2, 25 November; D3, 5 December; D4, 15 December; D5, 25 December.

### 3.4 Stress identification and evaluation for adaptation

**Figure 5:**
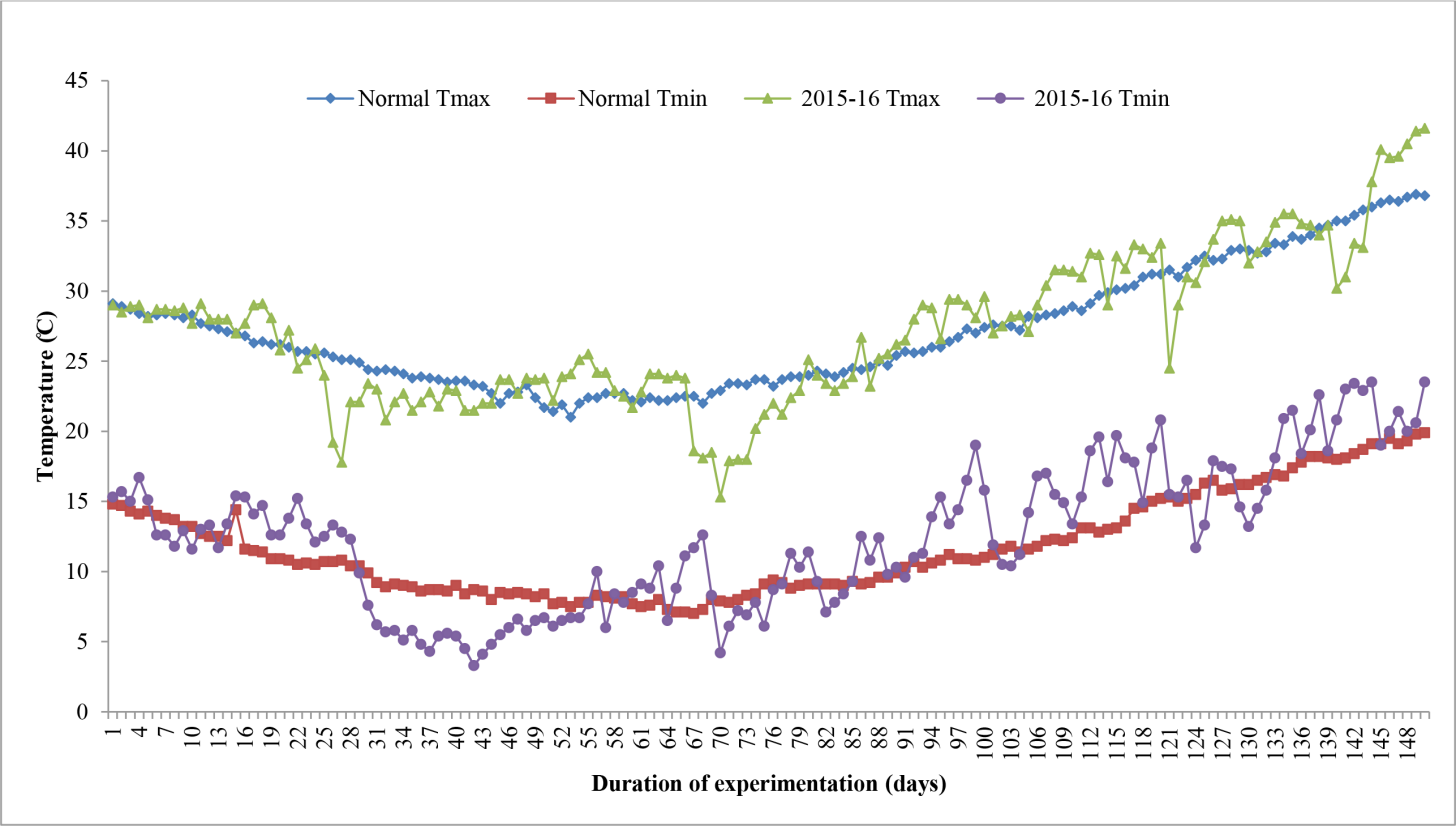
Variation of actual maximum temperature (Tmax) and minimum temperature (Tmin) during wheat growing seasons of 2015–2016 along with normal.

The sums of deviation from optimum thresholds of maximum temperature and minimum temperature for sensitive growth phases, viz., flowering to milking and flowering to maturity, were correlated with grain filling duration and grain yield to identify the degree of association and impact of heat stress. Data revealed that the sum of deviation from threshold maximum temperature (tTmax) for the F-Mlk period varied between 3.4°C in D1 in 2014–2015 and 59.5°C in D4 in 2015–2016 (**Figure 2**). The highest sum of deviation from tTmax for the F-Mlk period for 2014–2015, 2015–2016, and 2016–2017 was recorded with D5 (29.8°C), D4 (59.5°C), and D4 (45.8°C), respectively. Late sowing (D4 and D5) had a higher sum of deviation from tTmax during F-Mlk than the crop sown on D1 or D2 or D3 in general. The sum of deviation from threshold minimum temperature (tTmin) for the F-Mlk period varied between −12.1°C in D1 for 2016–2017 and 47.9°C in D5 for 2015–2016 (**Figure 2**). The highest sum of deviation from tTmin for the F-Mlk period for 2014, 2015, and 2016 was recorded with D3 (31.0°C), D5 (47.9°C), and D4 (18.6°C), respectively. The sum of deviation from tTmax for the F-Mat period varied from 47.6°C in D1 in 2014 to 232.5°C for D5 in 2015 (**Figure 2**). The highest sum of deviation from tTmax for the F-Mat period for 2014–2015, 2015–2016, and 2016–2017 was recorded with D5 (146.8°C), D5 (232.5°C), and D4 (162.2°C), respectively. Delaying the sowing operation increased the magnitude of the sum of deviation from tTmax for the F-Mat period. Late sowing (D4 and D5) had a higher sum of deviation from tTmax during F-Mat than the crop sown on D1 or D2 or D3. The sum of deviation from threshold minimum temperature (tTmin) for the F-Mat period varied between −49.9°C in D1 for 2016–2017 and 117.9°C in D5 for 2015–2016 (**Figure 2**). During the wheat growing season of 2008–2009, the accumulated sum of deviation from normal was negatively associated with the grain yield of wheat, and the number of tillers per m^2^ was reduced by 30%–35% due to high-temperature stress, lowering grain yield by 25%–30% (Anonymous, 2009). The highest sum of deviation from tTmin for the F-Mat period for 2014–2015, 2015–2016, and 2016–2017 was recorded with D5 (56.9°C, 117.9°C, and 95.4°C, respectively). Like tTmax during F-Mat, late sowing (D4 and D5) produced a higher sum of deviation from tTmin during F-Mat than sowing on D1, D2, or D3. The higher sum of deviation from thresholds due to the occurrence of high temperature during the post-heading period as a result of late sowing tends to accelerate the crop senescence and consequently shorten the duration (Dias and Lidon, 2009; Talukder et al., 2014). The greater the sum of deviation, the higher the heat stress experienced by the crop. This caused grain yield to reduce for later sown crops (beyond 25 November). At sensitive growth stages, such as flowering to milking, greater head load as manifested by the higher accumulation of sum of deviation (SD) enhanced canopy temperature (Siebert and Ewert, 2014), disrupted pollination, and caused pollen sterility, reducing the number of grains, consequently leading to reduced grain yield (Wheeler, 2012; Tao et al., 2015). The plots of maximum and minimum temperatures against the normal during three wheat growing seasons of the experimental period indicated that high temperatures during the reproductive and grain-filling period of wheat led to poor yield and yield attributes during 2015–2016 (**Figure 5**). For comparison with other years, maximum and minimum temperatures against the normal values are given in **Supplementary Figure 1**.

**Figure 6:**
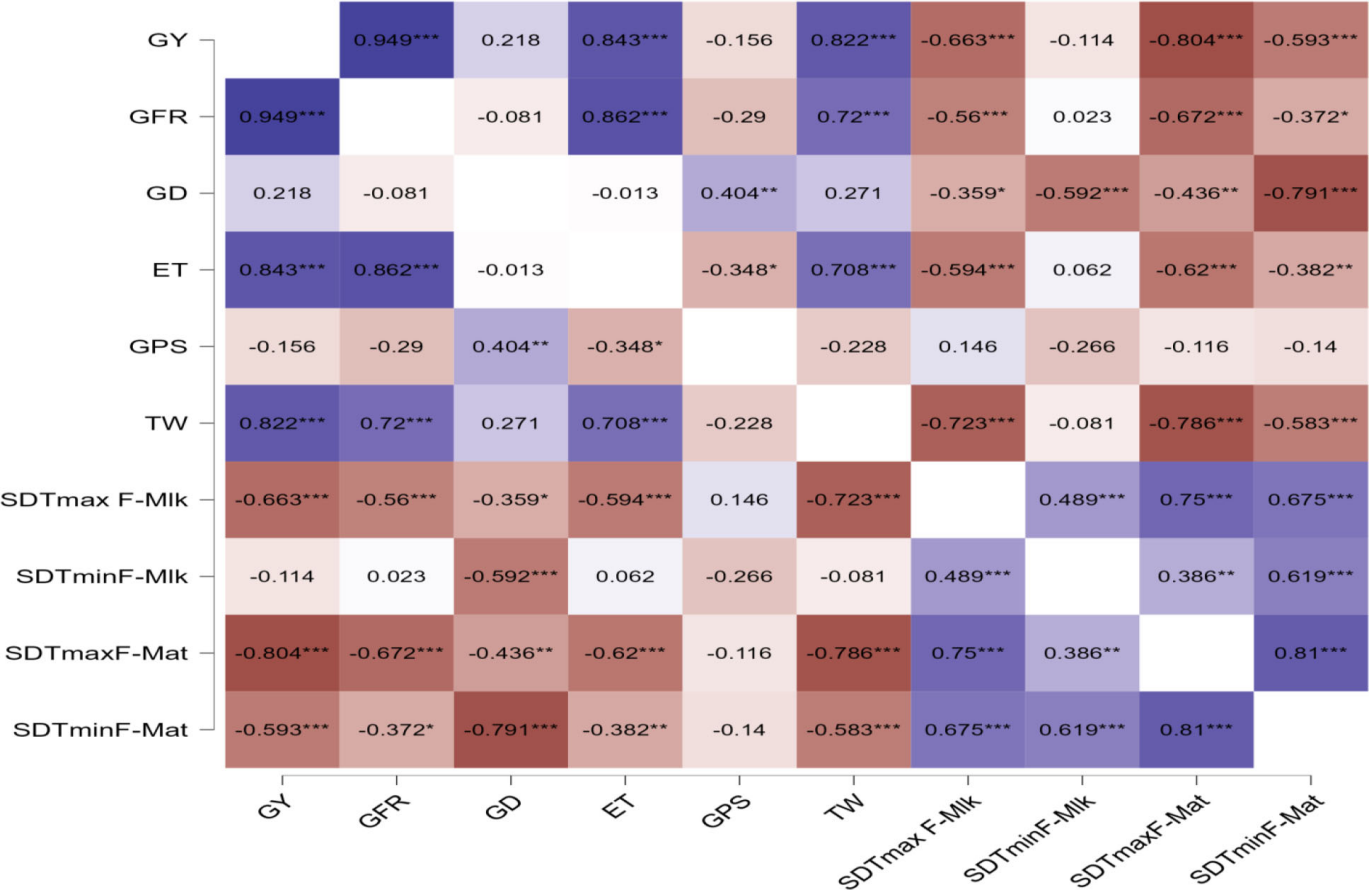
Correlation between grain yield, grain filling duration, and its rate and yield attributes with sum of deviations from threshold maximum and minimum temperature from flowering to milking stage and flowering to maturity stage in wheat. * p< 0.05, ** p< 0.01, *** p< 0.001. GY, grain yield; GFR, grain filling rate; GD, grain filling duration; ET, number of effective tillers per m^2^; GPS, number of grains per spike; TW, test weight; tTmax, threshold maximum temperature; tTmin, threshold minimum temperature; SDTmaxF-Mlk, sum of deviation from tTmax for F-Mlk; SDTminF-Mlk, sum of deviation from tTmin from F-Mlk; SDTmaxF-Mat, sum of deviation from tTmax for F-Mat; SDTminF-Mat, sum of deviation from tTmin from F-Mat.

The correlation between grain yield, grain filling duration, and its rate and yield attributes with the sum of deviations from threshold maximum and minimum temperature from flowering to milking stage and flowering to maturity stage in wheat is presented in **Figure 6**. The correlation study indicated that grain yield was negatively and significantly affected by the sum of deviation (SD) from tTmax for F-Mat (SDTmaxF-Mat) (−0.804***) and the sum of deviation from tTmax for F-Mlk (SDTmaxF-Mlk) (−0.663***) followed by the sum of deviation from tTmin from F-Mat (SDTminF-Mat) (−0.593***). Sattar et al. (2020) observed that maximum temperature, minimum temperature, and bright sunshine hour occurring during 50% flowering to milking and 50% flowering to maturity phases of wheat demonstrated a negative correlation with grain yield.

Similarly, SDTminF-Mat (−0.791***), the sum of deviation from tTmin from F-Mlk (SDTminF-Mlk) (0.592***), SDTmaxF-Mat (−0.436**), and SDTmaxF-Mlk (−0.359*) negatively and significantly impacted the grain filling duration. SDTmaxF-Mat (−0.672***), SDTmaxF-Mlk (−0.56***), and SDTminF-Mat (−0.372*) negatively and significantly impacted the grain filling rate. Liu et al. (2018) observed a significant negative correlation of the phase duration of wheat with mean temperature. Effective tillers per m^2^, which positively and significantly affected GY (0.843***), were negatively and significantly affected by SDTmaxF-Mat (−0.62***), SDTmaxF-Mlk (0.594***), and SDTminF-Mat (−0.382**). Test weight, which positively and significantly affected GY (0.822**), was negatively and significantly affected by SDTmaxF-Mat (−0.786***), SDTmaxF-Mlk (−0.723***), and SDTminF-Mat (−0.583***). However, the number of grains per spike did not vary significantly by these deviations SD from tTmax and tTmin, implying that this plant parameter is genetic and is not affected by temperature variation. Arduni et al. (2009) hypothesized that day length affects spikelet initiation and number. From the present study, it was revealed that SDTmaxF-Mat, SDTmaxF-Mlk, and SDTminF-Mat negatively impact the number of effective tillers per m^2^, which is one of the most important yield attributes of wheat. Similarly, SDTmaxF-Mat, SDTmaxF-Mlk, and SDTminF-Mat negatively impact test weight and grain filling rate. Similarly, SDTmaxF-Mat, SDTmaxF-Mlk, SDTminF-Mat, and SDTminF-Mlk negatively impact grain filling duration. The strategy should be such that grain filling duration is completed before the onset of critical temperature thresholds of 29°C–30°C (Dubey et al., 2019; Sattar and Srivastava, 2021). In a study conducted by Hatfield et al. (2011), cereal grain yield was found to decrease between 4.1% and 10% due to an increase in the seasonal average temperature by 1°C. In the present study, excess thermal load computed in terms of the cumulative sum of deviation from thresholds provided an important criterion for assessing the impact of thermal stress on crop yield. Tao et al. (2017) highlighted the importance of different impacts of maximum and minimum temperatures during different growth stages of winter wheat, as well as the importance of management (e.g., shift of sowing date) and cultivars’ shift in adapting to climate change in the major wheat growing region.

### 3.5 Simulating the future yield of wheat by CERES-wheat model

**Figure 7:**
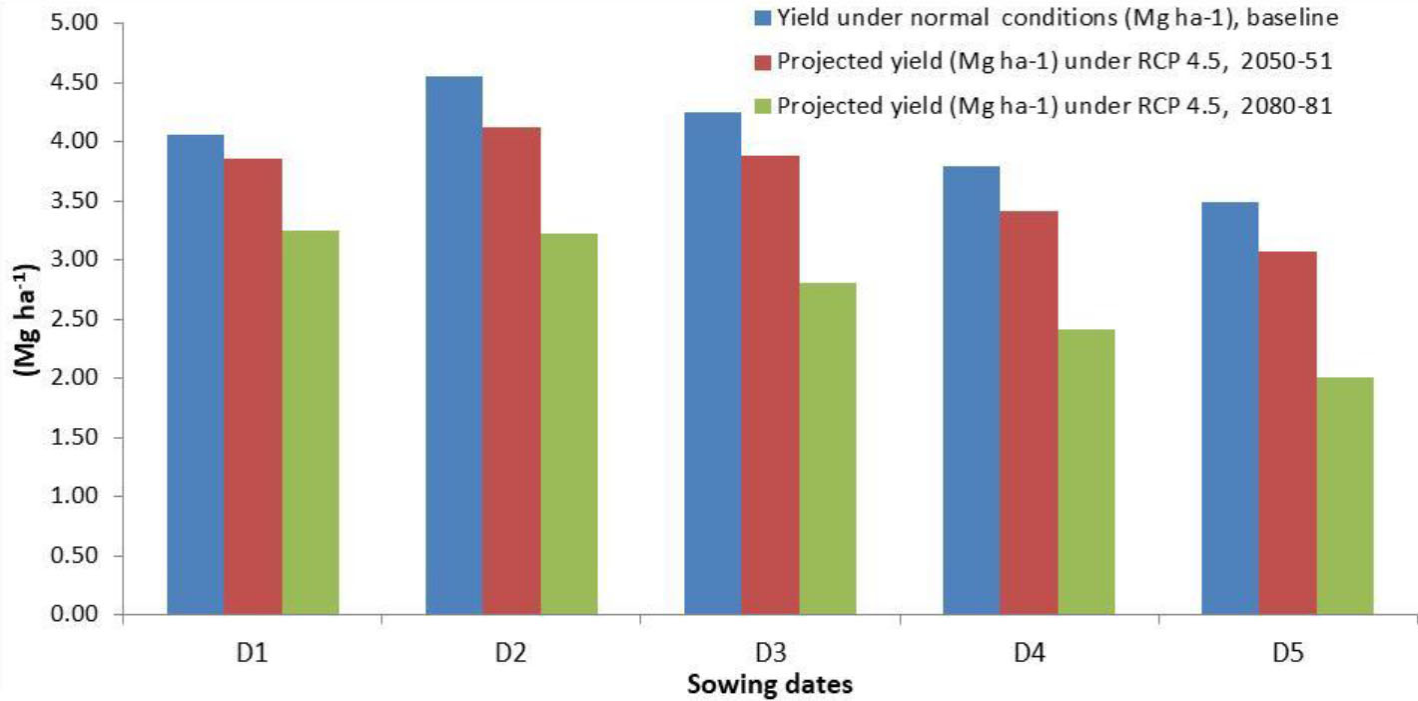
Predicted yield of wheat for 2050-51 and 2080-81 on different sowing dates by CERES-Wheat model under RCP 4.5. D1, 15 November; D2, 25 November; D3, 5 December; D4, 15 December; D5, 25 December.

**Figure 8:**
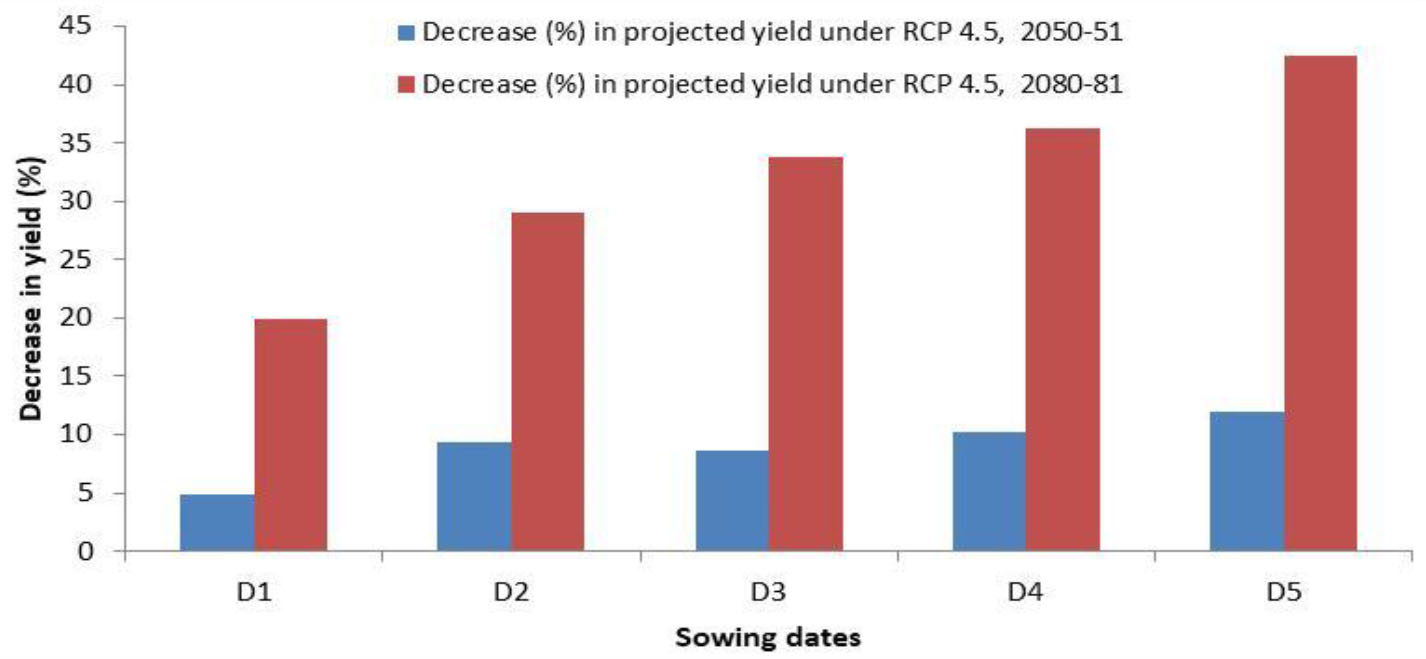
Decrease (%) in wheat yield in 2050-51 and 2080-81 on different sowing dates by CERES-Wheat model under RCP 4.5. D1, 15 November; D2, 25 November; D3, 5 December; D4, 15 December; D5, 25 December.

The projected yields of wheat for the year 2050-51 and 2080-81 simulated through the CERES-wheat DSSAT model is presented in **Figure 7**, which revealed that during 2050-51, the predicted wheat yield will vary from 3.07 to 3.88 Mg ha^−1^ across different sowing dates from 15 November to 25 December. Considering the projected yield for 2080-81, a significant decrease was observed, and it is predicted to range between 2.01 and 3.25 Mg ha^−1^. For the crop planted during November and December, a decrease in yield by 4.9% to 12.0% and 33.8% to 42.4% is predicted during 2050-51 and 2080-81, respectively (**Figure 8**). Chhabra and Haris (2014) also reported a decline in wheat yield in the region by 3.6%–13% in 2050 and 14.1%–40% in 2080. The grain yield of wheat was projected to decline in Pakistan by 7%–18% in 2050 and 9%–30% in 2090 under RCP 4.5 (Ishaque et al., 2023). In view of the significant decrease in wheat yield in the future, effective mitigation and adaptation measures will be required to sustain wheat production in the region.

## 4 Conclusion

The responses of phenology, yield, and yield attributing characters of wheat to differential thermal regimes are critically evaluated in this article, and we found that the crop response did vary significantly across different sowing environments. Excess thermal load computed in terms of the cumulative sum of deviation from critical thresholds provided a useful criterion for assessing the effect of heat stress on wheat yield in association with various agrometeorological indices such as GDDs, HTU, and accumulated PTU. Differential sowing dates generated varied weather patterns especially in terms of the thermal requirement during the growing period of wheat. For a higher yield of wheat (>4.0 Mg ha^−1^) in the region, it is necessary that grain filling is completed by 15 March, beyond which yield decreases substantially (0.5 Mg ha^−1^ per week) due to high-temperature stress. The optimal and sub-optimal conditions for wheat growth stressed the importance of the manipulation of sowing dates. Hence, it is necessary that wheat is planted at appropriate times for higher productivity. Critical responses of wheat phenology, yield, and yield attributing characters to varying sowing environments indicated that tactical decisions by the wheat growers keeping heat stress in mind would form an important guiding factor for wheat farming. Heat stress during the post-heading period is a serious climatic constraint for successful wheat production in the region. Since wheat growth is very sensitive to temperature, farmers in the area would be advised to finish their wheat planting before 25 November. Accordingly, shifting the planting time from the window of 25 November–10 December, which is usually practiced by 80% of wheat growers of the region to the window as prescribed in this study (i.e., finishing wheat sowing before 25 November), would be an important adaptation option for realizing higher yield and mitigating the negative impact of terminal heat stress on wheat growth and productivity. GGE biplot analysis indicated that RAU-3711 performed better when sown on 25 November (D2), whereas HD-2824 and HD-2733 performed better under 5 December (D3) and 15 December (D4) and 15 November (D1) and 25 December (D5). Wheat yield is predicted to decline significantly in 2050-51 and 2080-81 under RCP 4.5 scenario. Further studies using different models with a range of cultivars and management practices are needed to evaluate the impact of future climate change on wheat yield in the region.

## Data availability statement

The original contributions presented in the study are included in the article/**Supplementary Material**. Further inquiries can be directed to the corresponding author.

## Author contributions

Conceptualization: AS. Methodology: AS, GN, SKB. Data curation: AS, GN, GS, SKB. Original draft: AS, GN. Writing, review, and editing: AS, GN, RKJ, SKB, GS. Visualization: AS, GN. All authors contributed to the article and approved the submitted version.
