# Supplementary material for: Responses of phenology, yield attributes, and yield of wheat varieties under different sowing times in Indo-Gangetic Plains
Source: Front Plant Sci. 2023 Jul 21;14:1224334. doi: 10.3389/fpls.2023.1224334 (PMC10401057; doi:10.3389/fpls.2023.1224334)
Supplement: Supplementary file 1 [file DataSheet_1.docx]

**Supplementary table 1:**Yield and yield attributes of wheat as affected by year, sowing environment and cultivars.

| Year (Y) | Grain yield (Mg ha^-1^) | Effective tillers per m^-2^ | No. of grains per spike | Test weight (g) |
| --- | --- | --- | --- | --- |
| 2014-15 | 4.03a | 489.3a | 42.9b | 40.7a |
| 2015-16 | 1.96c | 285.6b | 43.9b | 30.4b |
| 2016-17 | 2.47b | 291.7b | 51.5a | 31.4b |
| Date of Sowing (D) |  |  |  |  |
| D1 | 3.03b | 366.1a | 47.3a | 37.2a |
| D2 | 3.21a | 367.1a | 44.8b | 37.1a |
| D3 | 3.04ab | 352.1ab | 47.6a | 35.0a |
| D4 | 2.65c | 313.9c | 46.1ab | 32.5b |
| D5 | 2.16d | 328.5bc | 44.7b | 29.1c |
| Cultivars (C) | |  |  |  |
| RAU-3711 | 2.78 | 330.5b | 50.2 | 33.62 |
| HD-2824 | 2.84 | 336.0b | 44.9 | 34.50 |
| HD-2733 | 2.84 | 370.2a | 43.2 | 34.36 |

Values with atleast a common letter down the column are not significantly different from each other according to LSD test (p<0.05). D1- 15 November; D2- 25 November; D3- 5 December; D4- 15 December; D5- 25 December.

**Supplementary fig. 1.** Variation of actual maximum temperature (Tmax) and minimum temperature (Tmin) during wheat growing seasons of 2014-15, 2015-16 and 2016-17 compared to their normal.
